# Feeding Dimorphism in a Mycophagous Nematode, *Bursaphelenchus sinensis*

**DOI:** 10.1038/s41598-019-50462-z

**Published:** 2019-09-27

**Authors:** Natsumi Kanzaki, Taisuke Ekino, Robin M. Giblin-Davis

**Affiliations:** 1 Kansai Research Center, Forestry and Forest Products Research Institute (FFPRI), 68 Nagaikyutaroh, Momoyama, Fushimi, Kyoto 612-0855 Japan; 2 Fort Lauderdale Research and Education Center, Department of Entomology and Nematology, University of Florida/IFAS, 3205 College Avenue, Davie, FL 33314 USA; 3 Department of Applied Biological Sciences, Saga University, Saga, 840-8502 Japan; 4 The United Graduate School of Agricultural Sciences, Kagoshima University, Kagoshima, 890-0065 Japan; 5 Present Address: School of Agriculture, Meiji University, Kawasaki, Kanagawa 214-8571 Japan

**Keywords:** Developmental biology, Ecology, Zoology

## Abstract

Phenotypic plasticity has been widely reported in animals and can drive investment in new biological characters that engender ecological adaptability. The nematode family Diplogastridae, especially *Pristionchus pacificus* with its dramatic stomatal (feeding) dimorphism, has become an important model system to analyze the evolutionary and developmental aspects of polyphenism. However, this plasticity has not been confirmed in other nematode groups. In the present study, we experimentally examined the feeding dimorphism of a fungal feeding free-living nematode, *Bursaphelenchus sinensis*. In a laboratory culturing experiment, the nematode expressed dimorphism, i.e., a small proportion of the population manifested as a predatory form. This form only occurred in females and was not clearly influenced by the presence of potential prey species. In addition, the ratio of the predatory form to the mycophagous form varied among different fungal food species grown in monoculture on different culture media. The predatory form of *B*. *sinensis* was typologically similar to the monomorphic (specialized) predators belonging to the same family. However, some essential morphological characters were slightly different from the specialized predators, and their behaviours were clearly disparate, suggesting that predation in *B*. *sinensis* is derived from a different phylogenetic origin than that of the specialized predators.

## Introduction

Phenotypic plasticity, morphological and functional polymorphism without genetic alteration, is an interesting biological phenomenon that pertains to the evolution of new characters for successful ecological adaptation^1–5^.

This phenomenon is widely found in animals, e.g., the body size and mouthparts of some tadpoles can differ according to environmental conditions^6,7^, and the body size, colour, and wing length of some locusts can change according to population density^8^. This developmental plasticity is also known in nematodes, one of the most important metazoan model/satellite model systems^9^. Stomatal (feeding) dimorphism/polymorphism is well known in the family Diplogastridae, a group of mostly bacteria feeding nematodes^10^, and a key member of the family, *Pristionchus pacificus* has become a model system for studying this phenomenon because of its short life cycle, ease of experimental manipulation, and conspicuous dimorphism^11–13^. In *P*. *pacificus*, the predatory (eurystomatous) form appears in microbial feeding populations (stenostomatous mouth form) when the culture ages or when co-cultured with a potential nematode prey species^14,15^. The genetic mechanism and chemical cues have been elucidated and involve a sulfatase switch gene acting in a dose-dependent fashion in concert with a newly discovered sulfotransferase gene^16–19^.

Stomatal phenotypic plasticity has not been experimentally examined in any other groups of nematodes even though it has been suspected from field observations on several *Bursaphelenchus* species. Almost all species in the genus are fungal feeders with a few facultative and obligate plant parasites. *Bursaphelenchus* spp. commonly have a syringe-like feeding structure referred to as ‘stylet’ to insert into fungal and plant tissue for extracting nutrients. Braasch and Braasch-Bidasak^20^ reported a ‘parasitic form’ of *B*. *aberrans* which has a flattened head and longer stylet, and considered it to be an insect-parasitic form of the species. Also, Kanzaki *et al*.^21,22^ reported a ‘parasitic adult form’ in *B*. *luxuriosae* and *B*. *doui* which do not have a stylet and are recovered from the body cavity and tracheal system of their respective cerambycid beetle hosts. However, the parasitic form of *B*. *aberrans* has only been reported from field-collected materials, its species status has not been confirmed with genotyping, and its insect parasitism or feeding behaviour have not been experimentally observed. Also, the parasitic forms of the other two species were recently confirmed as an alternative phoretic form, i.e., a non-feeding transportation survival form^23^. Therefore, the presence/absence of functional feeding dimorphism/polymorphism has not been confirmed yet in non-diplogastrid nematodes.

In the present study, we conducted experiments to elucidate the feeding (stylet) dimorphism of a cultured isogenic (single female) isolate of the mycophagous nematode, *B*. *sinensis*, which is a close relative of (or possibly conspecific with) *B*. *aberrans*^24,25^.

## Results

### Occurrence of predatory form

In the preliminary experiment, the predatory form was only observed in adult females. The average ratios of the predatory form to the mycophagous form in the monoculture treatment were 10.1, 7.0 and 4.8% at 10, 20 and 30 days after inoculation, respectively. Whereas the ratios were 8.3, 5.9 and 8.4% in the prey (*Diplogasteroides asiaticus*) co-culture treatment (Supplementary Table S1). Because the number of replications was only three for each treatment, statistical analysis was not conducted for the ratios. However, the occurrence of the predatory form in monoculture on a commonly-used food fungus, grey mold (*Botrytis cinerea*), without potential prey was confirmed.

The predatory form was not observed in the other nine aphelenchoidid species regardless of the number and the population density of the nematodes (Supplementary Table S2).

### Morphological observation of predatory form

Morphometric values are summarized in Supplementary Table S3.

**Figure 1:**
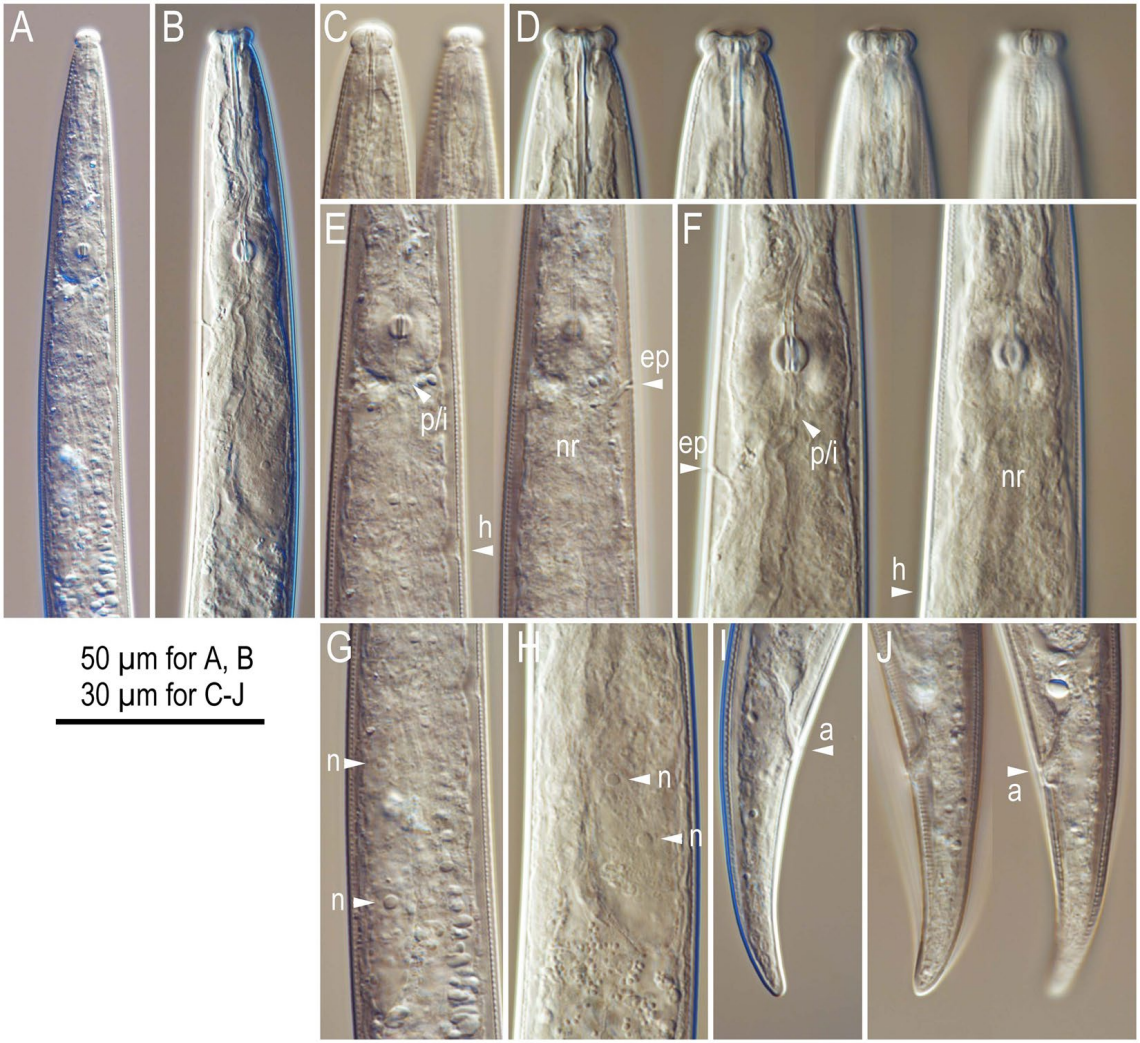
Comparisons between mycophagous and predatory-form females of *Bursaphelenchus sinensis*. (**A**,**B**) Anterior region of mycophagous (**A**) and predatory (**B**) forms; (**C**,**D**) Lip and stylet region of mycophagous (**C**) and predatory (**D**) forms in several different focal planes; (**E**,**F**) Metacorpus region of mycophagous (**E**) and predatory (**F**) forms in several different focal planes where “p/i”, “ep”, “h” and “nr” indicates pharyngo-intestinal junction, excretory pore, hemizonid and nerve ring, respectively; (**G,H**) Pharyngeal gland of mycophagous (**G**) and predatory (**H**) forms where “n” indicates nuclei of pharyngeal gland; (**I,J**) Tail of mycophagous (**I**) and predatory (**J**) forms where “a” indicates anal opening.

The predatory form occurred only in adult females, and the posterior part of their bodies were basically identical to that of the mycophagous form, which has been described previously^25,26^, i.e., the predatory form specific characters were observed only in the anterior region of the body (Figs 1, S1). Therefore, the full descriptions of both forms are provided as Supplementary Text 1, and only the anterior region of the predatory form is described here.

Head (lip region) is distinctly offset from body, separated by a clear constriction, forming crown-like shape in lateral view, i.e., square-shaped subventral and dorsal sectors protruding laterally, and the lip is ca three times as broad as high. Stylet possessing wide lumen comprising a conus that is a little less than half of total stylet length and a shaft with large and clear basal swellings. The stylet lumen opens ventrally like the tip of an injection needle. Procorpus is cylindrical, short, less than 1.5 stylet lengths (=metacorpal lengths) long, ending in well-developed muscular metacorpus. Metacorpal valve is very prominent, and it is present at middle of metacorpus. Dorsal pharyngeal gland orifice opens into lumen of metacorpus mid-way between anterior end of metacorpal valve and anterior end of metacorpus. Pharyngo-intestinal junction is located immediately posterior to metacorpus. Dorsal pharyngeal glands are wide and short, i.e., more than half of corresponding body diam., and rather short, i.e., less than three stylet (metacorpal) lengths long. The glands overlap the intestine dorsally.

### Culturing experiment

**Figure 2:**
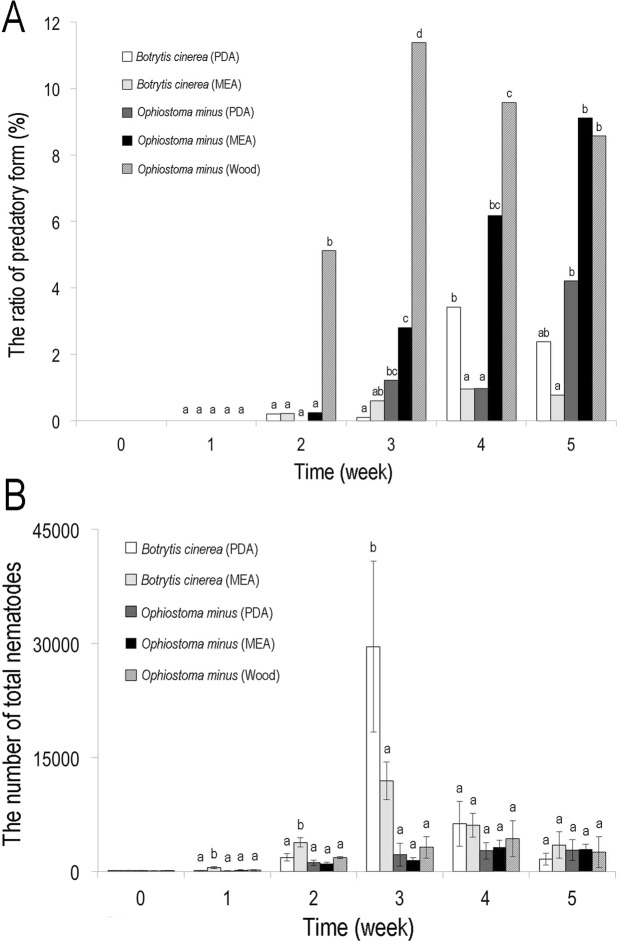
The ratio of predatory form (**A**) and the number of total nematodes (**B**) in five different culture conditions. The same letters indicate non-significant differences between the conditions at the same time point. Bars and error bars represent averages and standard deviations.

The results are summarized in Fig. 2 and Supplementary Table S4.

The predatory form occurred only in adult females, and no predatory-form male was observed during the experiment.

Predatory-form females occurred in all five treatments, i.e., *B*. *cinerea* on PDA and MEA and a blue-stain fungus, *Ophiostoma minus* on PDA, MEA and pine twigs. However, the ratio of the predatory form varied among treatments.

The ratio was highest in the pine twig-*O*. *minus* treatment where ca 5% of adult females manifested as the predatory form at two weeks after inoculation, then the ratio increased to ca 12% in the following week and later. The ratio was also relatively high in the MEA-*O*. *minus* treatment where the dimorphism was first observed at two weeks, and the ratio gradually increased to ca 9% at the end of experiment (five weeks). The ratios in the other three treatments were not high, i.e., the predatory form occurred in two or three weeks, and gradually increased to ca 3% in PDA-*B*. *cinerea* and PDA-*O*. *minus* treatments, or the ratio did not exceed 1% in MEA-*B*. *cinerea* treatment.

The nematode population growth also varied among the treatments and was relatively high in *B*. *cinerea* treatments. The nematodes rapidly increased for three weeks, and then rapidly decreased (population collapsed) in the PDA-*B*. *cinerea* treatment. The pattern was somewhat similar in the MEA-*B*. *cinerea* treatment, where the population was largest in the third week, and gradually decreased to the end of experiment. By contrast, the number of nematodes gradually increased in *O*. *minus* treatments, i.e., the population increased to ca 2000–4000 individuals in the third and fourth weeks and maintained at this level until the end of experiment.

### Observation of predation behaviour

**Figure 3:**
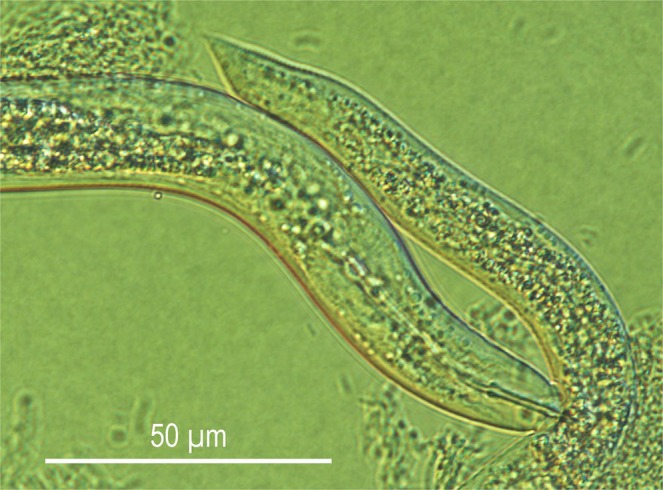
Predatory form-female of *Bursaphelenchus sinensis* feeding on *Acrobeloides* sp.

The predatory female successfully fed on *Acrobeloides* sp. (Fig. 3, Supplementary Video 1). *Bursaphelenchus sinensis* pierced the prey with its stylet and ingested the body contents by occasionally pumping the metacorpal valve. The predator occasionally wiggled its stylet during feeding by moving its head. The pumping and digesting (pharyngeal gland secretion) were not well-coordinated, i.e., pulsation of the anterior part of metacorpus (secretion) via the dorsal esophageal gland orifice often occurred during pumping.

### Culturing from predatory females

Immediately after the inoculation of predatory females and mycophagous males of *B*. *sinensis*, the predatory female attempted to feed on males, although it was not successful during our short-term observations. The females also fed on fungal hyphae. This feeding behaviour suggests that cannibalism can occur, and the predatory form is actually an omnivore, even if its stylet is morphologically specialized for predation.

Three days after inoculation, oviposition of predatory females was confirmed in four plates within five replications. The eggs all developed into mycophagous adults within 10 days.

## Discussion

### Feeding dimorphism in *B*. *sinensis*

First, the presence of feeding (stylet) dimorphism in *B*. *sinensis* was experimentally confirmed. This is the first case of an experimental study on the feeding dimorphism of a non-diplogastrid nematode.

The predatory form of *B*. *sinensis* has the following characteristics: (1) the form is not strongly induced by potential prey; (2) the form is found only in adult females, i.e., no predatory male was found during observations of more than 10,000 males; (3) the predatory adult female:mycophagous adult female ratio increases as cultures became older; (4) the ratio differed among media and fungal food species; and (5) the predatory form successfully fed on fungi, and produced mycophagous-form offspring. In addition, because the dimorphism was not observed in the other examined species, this phenomenon is considered to be a species (or intrageneric clade) specific apomorphy of *B*. *sinensis* (and its close relatives).

The increase of the predatory form ratio in older cultures and the variance among treatments suggest that the induction of the form is regulated by the accumulation of their own chemical signal(s) and starvation signal(s) from fungal food and environment. Generally, the predatory form ratio was higher in lower nutrient media (PDA is the richest, and twig is the poorest), and slowly growing fungi (*B*. *cinerea* grows faster than *O*. *minus*), suggesting that the food signal (less amount of food) enhances the development of the predatory form. This is in accordance with the population dynamics, i.e., populations on PDA (and *B*. *cinere*a) grow faster, and collapse after consuming and damaging the fungal food source. The gradual increase of the predatory female form also suggests that the accumulation of a nematode-derived signal substance(s) induce(s) the form, and the increase of this form from the third to the fourth week in the PDA-*B*. *cinerea* treatment may support this, i.e., the substance(s) have accumulated during the rapid population growth in the third week. In addition, a significantly high ratio of the predatory form in the pine twig-*O*. *minus* strongly suggests that the component(s) of the twig substrate enhance(s) the dimorphism directly or indirectly (via food fungus) as environmental signal(s).

This pattern seems analogous to the stomatal dimorphism of several diplogastrid species.

The eurystomatous (predatory) form occurs in pure cultures of each species with various ratios, and it is strongly induced by the presence of prey only in several genera (e.g., *Neodiplogaster*), i.e., the others (e.g., *Pristionchus*) are not affected by the presence of prey^15,27^. This suggests that the eurystomatous form is primarily induced by the accumulation of their own chemical signals^17–19^ and probably the decrease of chemical signals from their regular food source (bacteria in this case). Further, although detailed relationships among the culture period and the ratio of the eurystomatous form have not been reported, the form is often found in older cultures^15^, and the ratio differs among food sources^28^. In addition, female-biased dimorphism is also known in some diplogastrid species. For example, the eurystomatous male of *Allodiplogaster seani* is rare in cultured conditions, but its close relative, *A*. *josephi*, commonly produces a predatory male^29^. *Neodiplogaster acaloleptae*, where the dimorphism is enhanced by prey, produces eurystomatous males, although the ratio is lower than females^15^. We do not have a clear explanation for the physiological mechanism of this sex-dependent dimorphism. However, it seems ecologically adaptive for a lifeboat strategy because microbe-feeding males which do not attack other individuals could be a safe food source for predatory females to increase oviposition.

Currently, the chemical and molecular mechanisms of the eurystomatous form have been examined closely only in *P*. *pacificus*, and several small molecules derived from the nematode are known to induce the predatory morph^17–19^, although detailed information about the other signals has not been discovered yet.

No chemical analysis has been conducted for *B*. *sinensis* in the present study. However, based on the similarity in the above-mentioned biological characters, the induction of the predatory form of *B*. *sinensis* seems analogous to that of diplogastrid nematodes. Further comparative chemical and molecular analyses are necessary to elucidate the induction of the predatory form.

Interestingly, the dimorphism in diplogastrids usually occurs only in the stomatal region^10–15^, but the dimorphism in *B*. *sinensis* is observed not only in the lip and stylet region, but also in the pharyngeal glands which secret digestive enzyme. So far, the response(s) to chemical signal(s) and morphogenesis are the main focuses of the genetic analyses on diplogastrids^12,16–19^. Detailed analyses on ingestion/digestion-related genes in *B*. *sinensis*, and close comparison with those of diplogastrids may yield interesting parallels about the feeding plasticity of these nematodes.

### Functional morphology of predatory form

The predatory form has highly characteristic morphology, and is distinguished from the mycophagous form by a wide crown-like lip vs. small and rounded lip, long and thick stylet with wide lumen and large basal swellings vs. short and thin stylet with narrow lumen and small basal swellings, extremely well-developed metacorpus vs. small metacorpus, and wide and short (stout) pharyngeal glands vs. long and slender pharyngeal glands. These characters are also reflected in the morphometric values, e.g., size of lip, stylet and metacorpus (Supplementary Table S3). The composition of the feeding structure is rather similar to several aphelenchoidid predators, and these characters seemingly fit well in the “genus *Berntsenus*”. The genus was first established by Massey^30^, and then tentatively transferred to the insect parasitic/predatory subfamily Ektaphelenchinae as a genus *incertae sedis*^31,32^, i.e., the taxonomic/phylogenetic status of the genus is unclear. Based on the striking similarity with the predatory form of *B*. *sinensis* and the genus, *Berntsenus* is considered as an alternate form of *Bursaphelenchus*. However, the formal taxonomic revision will be presented elsewhere.

The predatory behaviour of several aphelenchoidid predators has been examined closely. Hechler^33^ and Kanzaki *et al*.^34^ examined the predation of *Seinura tenuicauda* and *S*. *caverna*, respectively, and Kanzaki^35^ reported the characteristic predation behaviour of the entomoparasite/predator species, *Ektaphelenchoides spondylis*. All three of these predators showed a well-orchestrated feeding behaviour, injecting a pharyngeal secretion (hypothesised to be a mixture of neurotoxin and digestive enzymes) for 1–2 min., and then sucking the body contents for ca 30 sec., i.e., the predators partly digested the prey externally before ingesting. In addition, the anterior part of the body is ventrally curved and only internal parts (metacorpus and pharyngo-intestinal valve) move during the feeding. By contrast, the feeding behaviour of the predatory form *B*. *sinensis* is not clearly coordinated, i.e., wiggling the head and stylet during feeding, and digestion and sucking occur simultaneously.

The crown-shaped lip region of the predatory form is somewhat similar to the lip structure of several dorylaimid and mononchid predators. These predatory species use their lips to search and confirm the availability of the prey^36^. Further, these predators use their crown-shaped lip region to grasp the prey to penetrate the cuticle (dorylaimids) or to aid in swallowing the prey body (mononchids)^36–39^, and this behaviour is not observed in other aphelenchoidid predators^33–35^. Therefore, it is possible that *B*. *sinensis* uses its wide crown-like lip region like dorylaimid predators. This is in accordance with the enlarged metacorpus in the predatory-form of *B*. *sinensis*, i.e., the dorylaimid nematodes feed upon the prey body using their strong pharyngeal muscles, and do not largely depend on extracorporeal digestion^36^. However, in the present study, although the feeding behaviour of *B*. *sinensis* was observed and video-captured, the prey-capturing behaviour was not clearly observed. More detailed observations on a cascade of feeding behaviours is necessary to understand the functional morphology of the predatory form.

*Bursaphelenchus* phylogenetically belongs to clade 4 of Aphelenchoididae, and is a sister to the clade of insect parasites and predators (clade 3) (Supplementary Fig. S3). However, because of the differences in predatory behaviour, the predation in clade 3 and *B*. *sinensis* are hypothesised to have evolved independently from each other. In addition, clade 3 predators have a glandular anterior 1/3 of metacorpus which is hypothesised to support injection of gland secretion(s), and a lack basal swellings of the stylet (providing connection for retractor muscles), although *B*. *sinensis* does not have this modification of the median bulb but possesses large basal swellings of the stylet. These structural differences corroborate independent evolutionary origins of predation.

Further genomic and transcriptomic studies will be necessary to confirm this hypothesis. In addition, the feeding dimorphism has been found only in *B*. *sinensis* in clade 4, and the presence/absence of this type of dimorphism in other aphelenchoidids will need to be examined to understand the ecological and evolutionary function of the phenomenon.

### Concluding remarks

Here, we experimentally confirmed the presence of the feeding dimorphism of *B*. *sinensis* and reported its basic morphological/biological characters. So far, this type of dimorphism, i.e., microbe feeding and omnivore (microbe feeding and predation) forms, has been found only in Diplogastridae which is a promising model system for the study of phenotypic plasticity^10,12,13^.

In addition to feeding dimorphism, Diplogastridae and Aphelenchoididae which includes *B*. *sinensis*, share many biological characters. For example, both families contain a variety of feeding modalities, i.e., Diplogastridae comprise mostly bacteria feeders, and secondarily evolved fungal feeders, insect parasites and predators^10^, and Aphelenchoididae which was originally mycophagous contains many obligate and facultative plant parasites, obligate and facultative insect parasites and predators^31,40^. Further, the majority of the members of both families utilize a variety of insects as phoretic and parasitic hosts. In other words, both families are associated with a wide variety of microbes, nematodes, insects and plants.

Although further studies are necessary to understand the genetic mechanisms, we expect that the family Aphelenchoididae will be a useful developmental and evolutionary comparative system to the family Diplogastridae.

## Materials and Methods

### Nematode culture

A population of *B*. *sinensis* was isolated from a dead Japanese red pine, *Pinus densiflora* planted at the experimental nursery in the campus of Forestry and Forest Products Research Institute, Tsukuba, Ibaraki, Japan (GPS: 36°00′21″N, 140°07′35″E, 25 m a.s.l.) in September, 2015. The nematodes were reared on a lawn of *B*. *cinerea* inoculated onto potato dextrose agar (PDA) medium. After the propagation of nematodes, gravid females were individually transferred to a *B*. *cinerea* lawn on 2.0% malt extract agar (MEA: 2.0% malt extract, 2.0% agar) to establish isogenic (single female) lines. The lines were confirmed for their species status based on the molecular sequence of small subunit ribosomal RNA, i.e., the sequences were identical to that of *B*. *sinensis* previously deposited in the GenBank database (AB232162). One of the isogenic lines coded as ‘BsIso1’ was used for the following experiments.

### Occurrence of predatory form in *B*. *sinensis*

First, we examined 1) the presence/absence of the feeding dimorphism and 2) the effect of co-culturing with a potential prey species on the dimorphism/polymorphism, i.e., we expected that the alternative morph is the predatory form as reported in diplogastrid species, if it is present. For this purpose, a culture experiment was conducted as a preliminary study. Because the ‘parasitic form’ of *B*. *aberrans* has been reported from field samples collected from dead pines, *P*. *kesiya*^20^, twigs of *P*. *densiflora*, the natural host of *B*. *sinensis* was used as a substrate.

Pine twigs (ca 7 mm diam. and 4 cm long) were autoclaved and individually transferred to a sterile plastic centrifuge tube (Falcon, 15 ml), and *O*. *minus* was inoculated onto them. A week after the fungal inoculation, 500 individuals of mixed-stage *B*. *sinensis* were inoculated onto each twig, and as a potential prey species, 500 mixed-stage individuals of *Diplogasteroides asiaticus*, which often cohabitate with *B*. *sinensis* in the dead pines^41,42^, were inoculated on half of the twigs.

After the nematode inoculation, twigs were incubated at 20 °C. The nematodes were recovered from the twigs every 10 days for 30 days. The recovered nematodes were heat-killed at 60 °C for 30 sec. and fixed in triethanolamine-formalin (TAF: 2.0% triethanolamine, 7.0% formalin, 91% distilled water). The ratio of stylet forms was examined for *B*. *sinensis* using fixed materials.

### Occurrence of predatory form in other aphelenchoidid nematodes

To examine the presence or absence of the dimorphism (predatory form) in other aphelenchoidid nematodes, nine aphelenchoidid species that were actually or potentially associated with coniferous trees, *B*. *xylophilus*, *B*. *luxuriosae*, *B*. *rainulfi*, *B*. *niphades*, *B*. *kiyoharai*, *B*. *poligraphi*, *B*. cf. *cryphali*, *B*. *wuae*, and *Aphelenchoides* sp.^43^, were examined for their feeding dimorphism. The nematodes were cultured on *O*. *minus* grown on pine twigs. the experimental conditions were the same as the above-mentioned preliminary experiment, i.e., 500 mixed-stage nematodes were inoculated onto the twig, and cultured for 20 days at 20 °C. The nematodes were then extracted from the twig, and the number of nematodes, nematode population density (number of nematodes/dry weight of twig), and the ratio of predatory form to total per 100 were examined. Three twigs were prepared for each species as replicates. For the examination of the predatory form, the first 100 males and females were examined for their feeding morphology under the light microscope. *B*. *sinensis* served as the positive control.

### Morphological observation of predatory form

The morphological characters of the predatory form were examined using the individuals successfully induced in the preliminary experiment. The fixed materials were processed in glycerine using a modified Seinhorst’s method^44^ and mounted in glycerine according to the methods of Maeseneer and d’Herde^45^. The mounted materials were observed for detailed morphological characters under light microscopy (Eclipse 80i; Nikon, Tokyo, Japan).

All photomicrographs were obtained using a digital camera system (MC170 HD; Leica, Wetzlar, Germany), and morphological drawings were made using a drawing tube connected to the microscope. The photomicrographs and drawings were edited with PhotoShop Elements 9 (Adobe) to adjust the contrast and brightness for constructing figures.

### Culture experiment

Because the presence of prey species did not clearly affect the occurrence of the predatory form of *B*. *sinensis*, the prey species was not used for the following culture experiments. To examine the effect of substrate (media) and food source (fungal species), we used three different media, PDA, MEA and *P*. *densiflora* twigs, and two different species of fungi, *B*. *cinerea* and *O*. *minus*.

Because *B*. *cinerea* does not grow well on pine twigs, the fungus was only inoculated onto PDA and MEA in 60 mm diam. plastic Petri dishes, whereas *O*. *minus* was inoculated onto both agar media and pine twigs (ca. 8 mm diam. and 8 cm long). The preparation of twigs was as described for the previous experiment.

After confirming fungal growth on each media type, 100 individuals of mixed-stage *B*. *sinensis* were inoculated onto each substrate and kept at 20 °C in the dark. The number of total nematodes and the ratio of the predatory form to the mycophagous form were examined every week after inoculation for five weeks. For the ratio of predatory forms, only adult nematodes were examined because the dimorphism only occurs in adult females. Five replicates were prepared for each treatment, i.e., 30 plates were prepared for each treatment, and five plates were harvested from each treatment every week.

### Observation of predatory behaviour

Predatory-form females were collected from the extra materials of the twig-*O*. *minus* treatment. The nematodes were transferred to a 2.0% water agar plate, and ca. 30 individuals of mixed-stage *Acrobeloides* sp., a bacterial feeding species which we regularly use as a standard food species for predatory nematodes^34^, were inoculated onto the plate. A cover slip was placed on the inoculated nematodes, and the plate was examined under the dissection microscope occasionally for one hour to observe feeding (predatory) behaviour. When the feeding behaviour was observed, it was closely observed and recorded under light microscopy using the digital camera system (MC170 HD).

### Examination of next generation of predatory females

Five to 10 predatory females collected from the twig-*O*. *minus* treatment were transferred to MEA previously inoculated with *B*. *cinerea*. In addition, 10 mycophagous males were transferred to the plate as the sperm (and potentially food) source for predatory females. The culture plates were observed under a dissection microscope every day for 10 days to examine the stylet form of the next generation adults and to test the fungal feeding ability of the predatory-form females.

### Statistical analysis

We identified significant differences in the total number of nematodes in the five different cultural conditions. Two-way analysis of variance test (substrates × fungi) with a Tukey honestly significant difference post hoc test was used for the multiple comparisons. To analyze the ratio of the predatory form to the mycophagous form under the different experimental conditions and because of the unevenness of the examined (available) female individuals among replications, i.e., we examined up to 200 individuals, but most nematodes were juveniles in many replications, and only less than 20 total females were available, all five replications were combined into a single data point. The ratio was compared using a chi-square test, and Ryan’s post hoc test was performed for multiple comparisons. All statistical analyses were performed with R ver 3.5.1^46^. P < 0.05 was considered to be significant.

## Supplementary information

Supplementary Information

Video
