# Supplementary material for: Feeding Dimorphism in a Mycophagous Nematode, Bursaphelenchus sinensis
Source: Sci Rep. 2019 Sep 27;9:13956. doi: 10.1038/s41598-019-50462-z (PMC6765002; doi:10.1038/s41598-019-50462-z)
Supplement: Supplementary file 1 — Supplementary Information [file 41598_2019_50462_MOESM1_ESM.pdf]

Supplementary Information

Feeding Dimorphism in a Mycophagous Nematode, *Bursaphelenchus sinensis*

Natsumi Kanzaki[1,2,*], Taisuke Ekino[3,4,5] and Robin M. Giblin-Davis[2]

[1]Kansai Research Center, Forestry and Forest Products Research Institute (FFPRI), 68 Nagaikyutaroh, Momoyama, Fushimi, Kyoto 612-0855, Japan

[2]Fort Lauderdale Research and Education Center, Department of Entomology and Nematology, University of Florida/IFAS, 3205 College Avenue, Davie, FL 33314, USA

[3]Department of Applied Biological Sciences, Saga University, Saga 840-8502, Japan

[4]The United Graduate School of Agricultural Sciences, Kagoshima University, Kagoshima 890-0065, Japan

[5]Present address: School of Agriculture, Meiji University, Kawasaki, Kanagawa 214-8571, Japan

*Corresponding. nkanzaki@ffpri.affrc.go.jp

Supplementary Text S1. Morphological description of *Bursaphelenchus sinensis*

The typological characters of the mycophagous form adults have already been described in previous studies. Therefore, some important characters to compare with those of the predatory form and close relatives, anterior part of body and tail region, are described here. The morphological characters were photomicrographed in Fig. 1 and drawn in Supplementary Fig. S1.

Mycophagous form adult

Cuticle thin, annulated, lateral field with two lines. Head distinctly offset from body, separated by a clear constriction, lip region in lateral view squarish rounded, *ca* twice as broad as high. Stylet with narrow lumen comprising a short cone *ca* one-third or slightly more of total stylet length and a shaft with small but clear basal swellings. Procorpus cylindrical, *ca* three stylet lengths (= metacorpal lengths) long, ending in well-developed metacorpus. Metacorpal valve clearly observed, present at middle of, or slightly posterior to, centre of metacorpus (median bulb). Dorsal pharyngeal gland orifice opening into lumen of metacorpus mid-way between anterior end of metacorpal valve and anterior end of metacorpus. Pharyngo-intestinal junction slightly posterior to metacorpus. Dorsal pharyngeal glands narrow, *i.e.*, less than one-third of corresponding body diam., *ca* five stylet (metacarpal) lengths, overlapping intestine dorsally. Nerve ring surrounding pharyngeal glands and intestine slightly posterior to pharyngo-intestinal junction. Secretory-excretory pore opening ventrally around the level of nerve ring. Hemizonid at *ca* one metacorpal length posterior to metacorpus, unclear in live material but distinct in permanently mounted material.

Predatory form adult

Predatory form was found only in females in the present study. Cuticle thin to moderate, coarsely annulated, lateral field with two lines. Head distinctly offset from body, separated by a

clear constriction, lip region in lateral view crown-like, *i.e.*, square-shaped subventral and dorsal sectors protrude laterally, *ca* three times as broad as high. Stylet with wide lumen comprising a conus which is a little less than half of total stylet length and a shaft with small but clear basal swellings. The stylet lumen opens ventrally like the tip of an injection needle. Procorpus cylindrical, less than 1.5 stylet lengths (= metacorpal lengths) long, ending in well-developed metacorpus. Metacorpal valve clearly observed, prominent, present at middle of metacorpus (median bulb). Dorsal pharyngeal gland orifice opening into lumen of metacorpus mid-way between anterior end of metacorpal valve and anterior end of metacorpus. Pharyngo-intestinal junction immediately posterior to metacorpus. Dorsal pharyngeal glands wide and short, *i.e.*, more than half of corresponding body diam., less than three stylet (metacorpal) lengths, overlapping intestine dorsally,. Nerve ring surrounding pharyngeal glands and intestine slightly posterior to pharyngo-intestinal junction. Secretory-excretory pore opening ventrally around the level of nerve ring. Hemizonid at *ca* 1.5 metacorpal lengths posterior to metacorpus, unclear in live material but distinct in permanent mounted material.

Male

Spicules, paired. Capitulum (rostrum + condyles) not well developed, forming ventrally inclined roundish rectangle where condyles and rostrum are not clearly distinctive. Dorsal limb of spicule (lamina) prominent, with almost straight anterior two-thirds and smoothly ventrally curved posterior one-third; posterior end with roundish-squared terminus without cucullus. Ventral limb (calomus) inconspicuous, forming triangular cuticle membrane. Gubernaculum or apophysis absent. Bursal flap present, narrow, tape-like, inconspicuous, starting from level of second paired genital papillae (P3) or posterior, not clearly observed from lateral aspect. Seven genital papillae: one ventral papilla (P1) and three ventral subventral paired papillae (P2, P3, P5) present: precloacal P1 papilliform, ventral, slightly anterior to cloacal opening (co); adcloacal P2 papilliform, on subventral body; P3 located mid-way between co and tail tip (tip of bursal flap) or slightly anterior; gland opening-like P5 on ventral side slightly posterior to mid-way between P3 and tail tip, possessing internal connection (unclear secretory duct-like structure).

Female

Vulval opening dome-shaped slit without clear vulval flap apparatus, but both sides of the anterior vulval lip slightly extend to cover both sides of vulval opening (side flap) which sometimes give an appearance of a short vulval flap. Tail conical; tail tip narrowly rounded to bluntly pointed.

Supplementary Fig. S1.

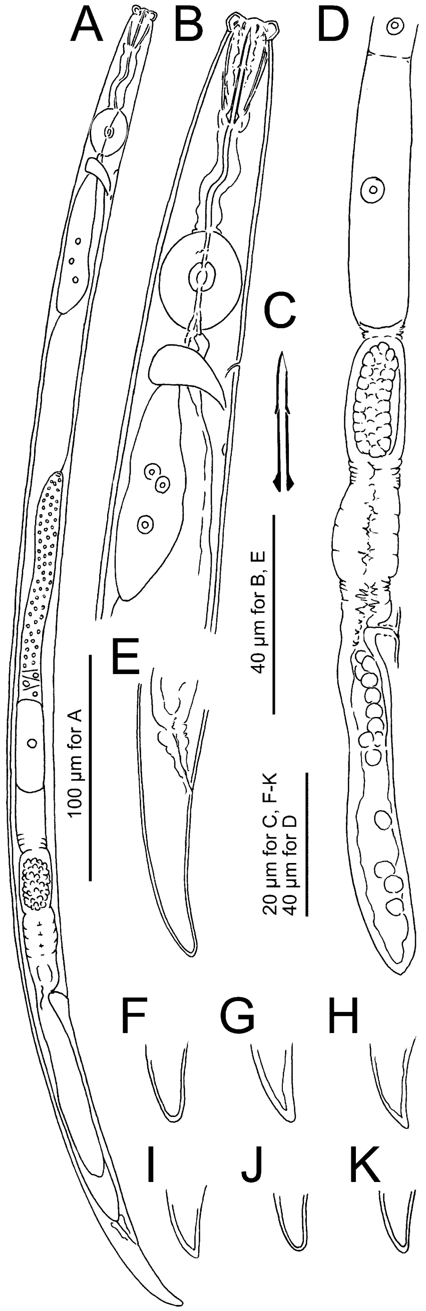

Figure S1. Predatory form female of *Bursaphelenchus sinensis* in right lateral view.

A: Entire body; B: Anterior region; C: Stylet; D: Posterior part of gonad; E: Tail; F-K: Variation in tail tip.

Supplementary Figure S2.

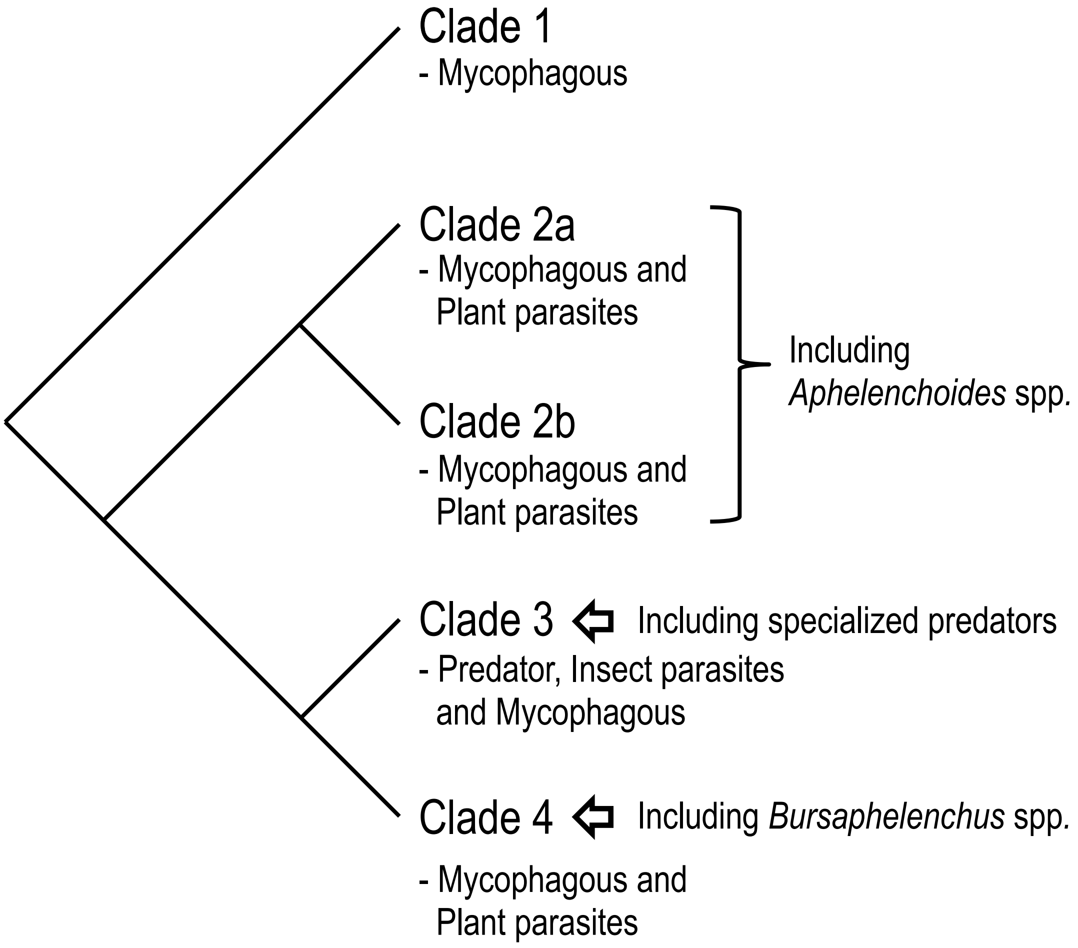

Figure S2. Schematic phylogenetic relationship within the family Aphelenchoididae. The species examined for the presence/absence of dimorphism belong to clades 2 (*Aphelenchoides* sp.) and 4 (*Bursaphelenchus* spp.)

Supplementary Table S1. Ratio of predatory form of *Bursaphelenchus sinensis* under two culture conditions, with or without co-culturing with *Diplogasteroides asiaticus* on pine twig. Data for three replicates are shown in %, and the mean is given in parentheses.

Inoculation period (days)	10	20	30
Pure culture male	0, 0, 0 (0)	0, 0, 0 (0)	0, 0, 0 (0)
Pure culture female	7.1, 9.3, 13.8 (10.1)	6.1, 6.3, 8.7 (7.0)	3.7, 4.3, 6.4 (4.8)
Co-culture male	0, 0, 0 (0)	0, 0, 0 (0)	0, 0, 0 (0)
Co-culture female	5.9, 8.4, 10.6 (8.3)	2.4, 6.2, 9.2 (5.9)	6.1, 6.7, 7.5 (8.4)

Supplementary Table S2. Examination of the occurrence of feeding dimorphism of other aphelenchoidid nematodes. The numbers are the average of three replicates.

Nematode species	Origin	Number of nematodes	Dry wood of twig	Nematode population density[1)]	Predatory-form female[2)]	Predatory-form male[2)]
Bursaphelenchus sinensis	*Pinus densiflora*	8633	2.56	3476	12	0
B. xylophilus	*Pinus densiflora*	29900	2.42	12360	0	0
B. luxuriosae	*Acalolepta luxuriosa*	27700	3.45	8192	0	0
B. rainulfi	*Dryocoetes uniseriatus*	15300	2.05	7228	0	0
B. niphades	*Niphades variegatus*	47267	2.35	20599	0	0
B. kiyoharai	*Xyleborus seriatus*	7600	3.07	2735	0	0
B. poligraphi	*Pinus thunbergii*	21200	3.08	6910	0	0
B. cf. *cryphali*	*Cryphalus piceae*	6900	3.13	2399	0	0
B. wuae	*Pinus armandii*	8233	2.17	3864	0	0
Aphelenchoides sp.	*Tomicus piniperda*	61933	2.93	22416	0	-[3)]

1) Number of nematodes / dry weight of substrate (twig).
2) Number of predatory form within 100 individuals.
3) *Aphelenchoides* sp. is seemingly a parthenogenetic species (no male was found).

Supplementary Table S3. Morphometrics of the voucher permanent-mounted materials of *Bursaphelenchus sinensis*. All measurements are in µm with the form, mean ± sd (range).

Measurements	Mycophagous male	Predatory female	Mycophagous female
n	10	10	10
L	557 ± 17 (530-580)	605 ± 37 (547-656)	580 ± 26 (545-629)
a	30.9 ± 1.9 (27.2-33.3)	27.1 ± 2.8 (22.3-30.3)	28.6 ± 2.1 (25.6-32.3)
b	9.9 ± 0.5 (9.0-10.5)	8.9 ± 0.5 (8.1-9.7)	10.1 ± 0.2 (9.8-10.5)
c	18.5 ± 1.2 (17.2-20.6)	17.0 ± 1.1 (15.4-19.3)	17.1 ± 0.7 (15.7-18.3)
c’	2.3 ± 0.2 (2.1-2.5)	3.4 ± 0.2 (3.1-3.7)	3.5 ± 0.2 (3.2-3.7)
T or V	63.5 ± 5.1 (52.8-69.5)	73.5 ± 0.8 (71.6-74.2)	73.2 ± 0.9 (71.4-74.5)
M	39.3 ± 1.1 (38.3-41.7)	44.6 ± 1.7 (42.1-47.4)	37.9 ± 2.2 (34.8-40.8)
Max. body diam.	18.1 ± 0.9 (16.9-19.7)	22.6 ± 2.7 (19.4-27.2)	20.3 ± 1.4 (18.4-21.9)
Lip diam.	5.9 ± 0.3 (5.5-6.2)	10.8 ± 0.3 (10.5-11.1)	5.8 ± 0.2 (5.5-6.2)
Lip height	3.1 ± 0.2 (2.7-3.5)	3.6 ± 0.2 (3.3-4.2)	3.0 ± 0.3 (2.5-3.5)
Lip diam. / height	1.9 ± 0.2 (1.6-2.0)	3.0 ± 0.2 (2.7-3.2)	2.0 ± 0.2 (1.7-2.2)
Stylet conus	4.6 ± 0.2 (4.5-5.0)	9.2 ± 0.5 (8.3-10.0)	4.5 ± 0.4 (4.0-5.0)
Stylet length	11.6 ± 0.2 (11.2-11.9)	20.6 ± 0.9 (19.4-22.2)	11.9 ± 0.4 (11.2-12.4)
Median bulb diam.	9.1 ± 0.4 (8.5-9.7)	14.6 ± 0.9 (13.3-16.1)	10.4 ± 0.8 (9.7-11.9)
Median bulb length	13.6 ± 0.9 (11.9-14.7)	19.9 ± 1.3 (17.8-21.7)	14.7 ± 0.9 (13.4-16.4)
Median bulb length / diam.	1.50 ± 0.1 (1.33-1.66)	1.36 ± 0.1 (1.21-1.50)	1.41 ± 0.1 (1.27-1.50)
Nerve ring[1)]	68 ± 2.5 (64-72)	74 ± 3.0 (71-80)	69 ± 3.0 (66-74)
Secretory-excretory pore[1)]	57 ± 3.9 (51-64)	74 ± 4.4 (68-82)	59 ± 5.1 (53-67)
Hemizonid[1)]	79 ± 3.7 (74-86)	85 ± 2.8 (81-90)	80 ± 4.0 (74-85)
Gonad length[2)]	354 ± 35 (279-398)	264 ± 46 (183-325)	245 ± 22 (195-268)
Cloacal or anal body diam.	13.1 ± 0.5 (11.9-13.4)	10.4 ± 0.5 (10.0-11.1)	9.8 ± 0.7 (9.2-11.2)
Tail length	30 ± 2.0 (27-34)	36 ± 2.0 (32-39)	34 ± 1.9 (31-37)
Spicule (arc)	20.3 ± 1.0 (18.3-21.2)	-	-

Spicule (chord)	16.5 ± 0.7 (15.1-17.3)	-	-
Vulval body diam.	-	20.6 ± 2.1 (18.3-23.9)	19.0 ± 1.1 (17.4-20.4)
Vulva-anus distance (VA)	-	125 ± 10.9 (112-144)	122 ± 6.2 (113-131)
Post-uterine sac length (PUS)	-	90 ± 11.4 (78-113)	89 ± 10.1 (67-102)
PUS/VBD	-	4.4 ± 0.5 (3.8-5.5)	4.7 ± 0.6 (3.3-5.1)
PUS% to V-A dist.	-	72.0 ± 7.6 (60.5-85.6)	73.2 ± 8.9 (51.3-84.3)

1) Distance from anterior end.
2) Entire gonad length including *vas deferens* for males, and length from vulval opening to the anterior tip of ovary (gonad length excluding the post-uterine branch) for female.

Supplementary Table S4. The ratio of predatory- form and the number of total nematodes in five different culture conditions. Values are given in a form, mean ± sd.

A. Ratio of the predatory form females to total females in %. All five replications were combined into a single data set.

Weeks after inoculation	0	1	2	3	4	5
PDA-*Botrytis cinerea*	0	0[a]	0.20[a]	0.10[a]	3.42[b]	2.38[ab]
MEA- *B. cinerea*	0	0[a]	0.21[a]	0.60[ab]	0.96[a]	0.78[a]
PDA-*Ophiostoma minus*	0	0[a]	0[a]	1.22[bc]	0.97[a]	4.21[b]
MEA-*O. minus*	0	0[a]	0.25[a]	2.80[c]	6.18[bc]	9.11[b]
Twig-*O. minus*	0	0[a]	5.13[b]	11.4[d]	9.58[c]	8.57[b]

Values within a column with the same characters are not significantly different (P = 0.05).

B. Number of total nematodes.

Weeks after inoculation	0	1	2	3	4	5
PDA-*B. cinerea*	100	106 ± 22[a]	1863 ± 473[a]	29580 ± 11233[b]	6264 ± 2954[a]	1644 ± 784[a]
MEA- *B. cinerea*	100	499 ± 132[b]	3828 ± 595[b]	11944 ± 2479[a]	6084 ± 1553[a]	3496 ± 1743[a]
PDA-*O. minus*	100	67 ± 37[a]	1156 ± 355[a]	2212 ± 1506[a]	2728 ± 1091[a]	2817.6 ± 1368[a]
MEA-*O. minus*	100	196 ± 48[a]	1056 ± 183[a]	1472 ± 364[a]	3216 ± 941[a]	2976 ± 613[a]
Twig-*O. minus*	100	175 ± 101[a]	1800 ± 130[a]	3180 ± 1413[a]	4312 ± 2344[a]	2544 ± 2040 [a]

Values within a column with the same characters are not significantly different (P = 0.05).
